# Supplementary material for: Collapsing retroviruses for efficient delivery of viro-toxic cargoes
Source: bioRxiv. 2026 Jun 29:2026.06.28.735095. Preprint. [Version 1] doi: 10.64898/2026.06.28.735095 (PMC13345315; doi:10.64898/2026.06.28.735095)
Supplement: Supplement 1 [file media-1.pdf]

Supplementary information for:

Collapsing retroviruses for efficient delivery of viro-toxic cargoes

Christopher D. Mullally[1], Bojana Stefanovska[1,2], Yanjun Chen[1], Harshita B. Gupta[1], Michael A. Carpenter[1,2], Reuben S. Harris[1,2,*]

[1] University of Texas San Antonio Health Sciences Center, San Antonio, TX, USA

[2] Howard Hughes Medical Institute, University of Texas San Antonio Health Sciences Center, San Antonio, TX, USA

Correspondence: rsh@uthscsa.edu

Supplementary Figure 1. Schematic of reverse transcription and second strand synthesis of CRV1.0

Supplementary Figure 2. Data from HeLa cells transduced with CRV2.0

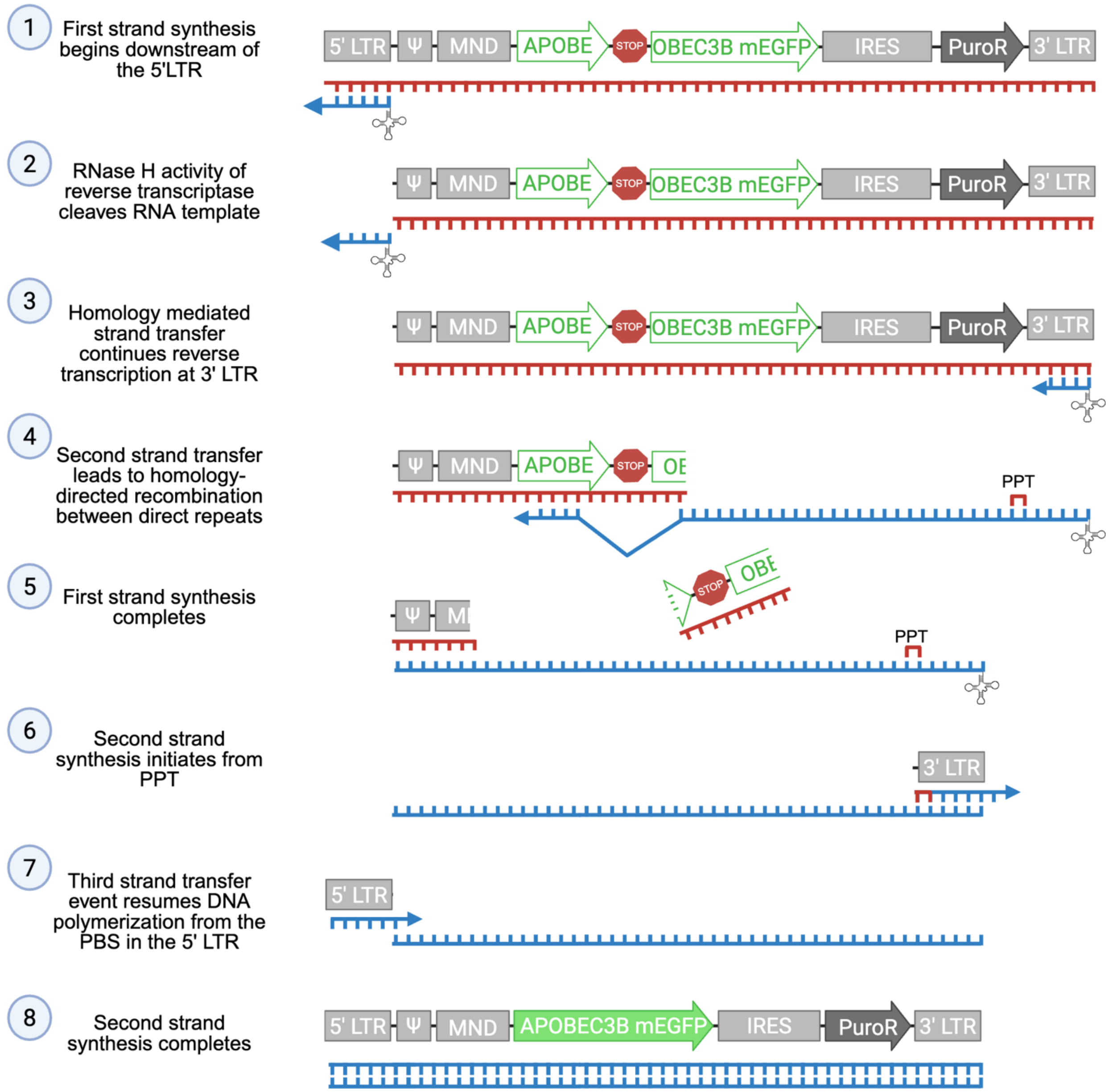
1
First strand synthesis begins downstream of the 5'LTR
2
RNase H activity of reverse transcriptase cleaves RNA template
3
Homology mediated strand transfer continues reverse transcription at 3' LTR
4
Second strand transfer leads to homology-directed recombination between direct repeats
5
First strand synthesis completes
6
Second strand synthesis initiates from PPT
7
Third strand transfer event resumes DNA polymerization from the PBS in the 5' LTR
8
Second strand synthesis completes
5' LTR
Ψ
MND
APOBE
STOP
OBEC3B mEGFP
IRES
PuroR
3' LTR
OBE
PPT
APOBEC3B mEGFP

Supplementary Figure 1. Schematic of reverse transcription and second strand synthesis of CRV1.0

RNA viral genome (red) is reverse transcribed into proviral DNA following the steps indicated. Both first- and second-strand synthesis require template switching events. CRV collapse is mediated by an additional template switch during first-strand synthesis. CRV2.0 constructs function similarly except P2A-T2A replaces the IRES.

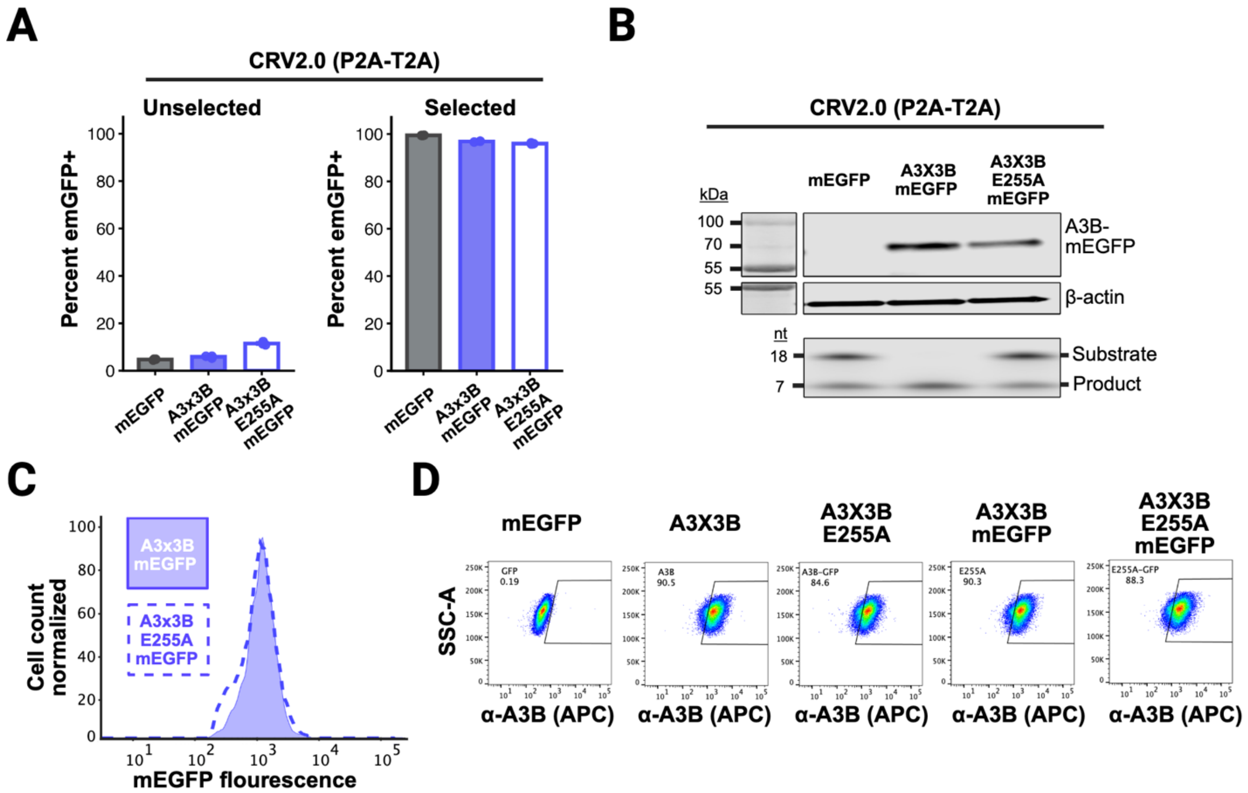
A
CRV2.0 (P2A-T2A)
Unselected
Selected
Percent emGFP+
mEGFP
A3x3B mEGFP
A3x3B E255A mEGFP
B
CRV2.0 (P2A-T2A)
mEGFP
A3X3B mEGFP
A3X3B E255A mEGFP
kDa
A3B-mEGFP
β-actin
nt
Substrate
Product
C
Cell count normalized
A3x3B mEGFP
A3x3B E255A mEGFP
mEGFP flourescence
D
mEGFP
A3X3B
A3X3B E255A
A3X3B mEGFP
A3X3B E255A mEGFP
SSC-A
α-A3B (APC)

Supplementary Figure 2. Data from HeLa cells transduced with CRV2.0

A, Quantification of percent mEGFP+ of HeLa cells transduced with CRV2.0 mEGFP, A3x3B-mEGFP, or A3x3B-E255A-mEGFP pre- and post-selection. Data represent the mean of three biological replicates ± SEM with individual datapoints jittered.

B, Immunoblots and DNA deamination cleavage assay results using HeLa cell lysates following transduction with the indicated CRV2.0 constructs (post-selection). Levels of A3B activity in CRV2.0 A3B-expressing cells are sufficient to promote full deamination of the 18nt oligo substrate, whereas the lower, residual deamination activity of the control reactions is likely due to endogenous A3B.

C, A3B- and A3B-E255A-mEGFP expression levels in live HeLa cells by flow cytometry [CRV2.0 A3x3B (blue) and A3x3B E255A (dashed blue)].

D, Flow cytometry dot plots of HeLa cells transduced with the indicated CRV2.0 and CRV2.1 constructs and stained with a specific anti-A3B monoclonal antibody.
